# Medical students’ perceptions of prosocial behaviors: a grounded theory study in China

**DOI:** 10.1186/s12909-024-05335-z

**Published:** 2024-03-29

**Authors:** Linya Jin, Tanisha Jowsey, Mei Yin

**Affiliations:** 1 School of Medical Humanity, Harbin Medical University, Nangang District, Heilongjiang Province, Harbin, China; 2 School of Medicine, Bond University, 14 University Drive, Robina, Gold Coast, QLD 4226 Australia

**Keywords:** Prosocial behavior, Medical students, Grounded theory

## Abstract

**Introduction:**

Prosocial behavior has been shown to be protective against burnout. Yet, we know little about prosocial behavior in medical students. We wanted to know what are chinese medical students' understanding of prosocial behavior and which factors influence their participation in it?

**Methods:**

We undertook a grounded theory study, following Corbin & Strauss. We used convenience sampling and conducted semi-structured individual interviews. We analyzed data using open, axial, and selective coding techniques. Next, we grouped data into concepts. We noticed these concepts aligned with three existing social theories, so we developed our theory in concert with these: the Theory of Planned Behavior, Self-Determination Theory, and Social Support Theory.

**Results:**

Twenty-eight medical students participated in this study. Medical students hold especial views on the roles of medical physicians, and most of these views align with students' core values, including the value of prosocial behavior. Students are intrinsically motivated to engage in prosocial behaviors that align with their core values. Personal values, personality traits, perceived self-competence, career motivation, environmental factors, and family influences are the core factors influencing medically positive prosocial behavior.

**Conclusions:**

This study supports a focus on prosocial behavior instead of altruistic behavior in medical education. We anticipate that promoting prosocial behavior through medical curricula will reduce moral distress and burnout among medical students.

**Supplementary Information:**

The online version contains supplementary material available at 10.1186/s12909-024-05335-z.

## Introduction

In the context of the COVID-19 pandemic, burnout among clinicians is at an all-time high [1–3]. Prosocial behavior is intended to benefit others. It is recognized in four types of practices: helping, comforting, sharing, and cooperating [4].” Prosocial behavior has been shown to be protective against burnout [5]. It has also been associated with improved individual job performance, career success, and career benefits, including higher job satisfaction, organizational commitment, and subjective assessments of job performance and career success [6].

Research on prosocial behavior in young adulthood has primarily focused on college students, measuring their levels of prosocial behavior [7], identifying factors that motivate prosocial behavior [8–14], and interventions [15]. Yet, we know little of medical student perceptions of prosocial behavior. This matters because while prosocial behavior has been recognized as protective against burnout, our understanding of its dynamics among medical students, particularly in the context of the COVID-19 pandemic, remains limited.

To date, medical education has focused on altruism, rather than prosocial behavior. Altruism is broadly understood as the selfless concern for the wellbeing of others and undertaking tasks to support others, even at expense to oneself. In China, this focus on altruism can be understood in the context that the Analects of Confucius (a philosopher and teacher Circa 500BCE), include attention to altruism as a virtue, and Confucian philosophy remains highly influential in Chinese society. Altruism is not the same as prosocial behavior, nor does it necessarily provoke prosocial behavior. Bateson and Powell explain,

*Prosocial behavior covers the broad range of actions intended to benefit one or more people other than oneself—actions such as helping, comforting, sharing, and cooperation. Altruism is motivation to increase another person's welfare; it is contrasted to egoism, the motivation to increase one's own welfare *[4]*.*

In 2007, British researchers Bishop and Rees called for a move in medical education away from altruism and toward prosocial behavior [16]. This call was echoed in 2012 by Burks and Kobus, whose literature review highlighted the dangers of emphasizing altruism in medical education [17]. While medical student perceptions of altruism remain in research focus [18, 19], there is a growing body of evidence that supports the call for emphasizing prosocial behavior. For example, prior to the pandemic, Ding et al.’s 2018 survey of medical students identified student motivations for engaging in prosocial behavior [14]. Students in this study were weary of altruism because of the associated risk of burnout. The authors recommend that medical curricula promote prosocial behavior rather than altruism in medical education. We identified two studies published in the pandemic context and focused on the prosocial behavior of medical students. During the COVID-19 pandemic, they reported on the behaviors and motivations of student volunteers. They showed that most students felt responsible for helping, had an open mind about prosocial behaviors, and suggested that clarifying roles and necessary skills could promote prosocial behaviors among medical students [20, 21]. Two further studies compared the differences in prosocial behaviors between medical students and students of other professions [22, 23]. Medical students and related disciplines showed high levels of prosocial behavior in both aid and comfort, with highly significant differences compared to students in sample professions not directly involved in such behavior. However, the healthcare industry is inherently helpful, a more appropriate scale has yet to be developed, and the measurements may be biased; therefore, there is a need for qualitative research on the prosocial behaviors of medical students. In a sample of Dutch university students, those studying medicine and psychology had more positive attitudes toward organ donation than their peers in economic sciences, with medical students scoring the highest [24–27]. Questions remain concerning which factors promote higher prosocial behavior among medical students.

In summary, there remains gaps in what we know about how medical students perceive prosocial behavior and which factors influence student participation in prosocial behavior. The quantitative research in this area is especially sparse, as is research within the pandemic context. We therefore undertook a grounded theory study in China among medical students from various specialties to explore their understanding of prosocial behavior and the factors that influence their engagement in prosocial behavior. Our research question was: what are Chinese medical students' understanding of prosocial behavior and which factors influence their participation in it?

## Methods

### Design

We undertook a grounded theory study, following Corbin and Strauss [28]. Grounded theory requires a systematic collection and analysis of data to derive a theory of what governs social behavior. We used convenience sampling and conducted semi-structured individual interviews.

### Recruitment

We conducted open recruitment on the social media platform ‘Xiaohongshu’ (https://www.xiaohongshu.com), where medical students are generally active. We contacted students via WeChat and conducted one-on-one semi-structured interviews via the Tencent meeting platform. Criterion for recruiting participants was current medical students. Through the medical education scenarios involved in the interviews, we could clarify the authenticity of the participants' identities. No requirements for grade level or clinical practice experience to allow for a broader collection of medical student perspectives. Participation involved a one-off interview and completion of a short demographic survey. Although it is common in grounded theory approaches to re-interview participants, we chose instead to iteratively build on our interview questions as interviews progressed. We compensated participants for their time with RMB 30.

### Data collection

We created a semi-structured interview guide in Chinese, which we developed based on the review of relevant literature (Appendix 1). Each participant was interviewed once. The general length of interviews was 45 min. All interviews were audio-recorded and transcribed verbatim by author [LJ]. Data collection and analysis were conducted iteratively. Each interview was summarized, and the written summaries were provided to participants for member checking. Interviews began with completion of a short survey to collect demographic data. The interviewer [LJ] took filed notes during interviews and these notes were also included as data.

All participants agreed on the interview content within summaries, and no changes were made. When participants’ quotes were used to illustrate results, participants were again approached to ask for their permission. Every participant agreed on the use of their quotes in this manuscript. Pseudonyms have been applied throughout.

### Data analysis

Before coding the survey descriptions, they were screened based on the following criteria: (1) clarity of description and (2) relevance of the description to the topic. LJ and MY completed this process and integrated similar descriptions for further analysis. The researchers read the transcripts and descriptions repeatedly to ensure their familiarity and sensitivity to the data in case important information was missed. We undertook three types of coding as described by Strauss and Corbin [28]: open, axial, and selective. The main steps include conceptualization and categorization, primary and secondary categories mining. LJ translated the transcripts into English, and authors from different cultures coded the transcripts through two rounds of inductive methods, using open coding to compare similar events and generate conceptual categories until thematic saturation was reached. The first round of coding was done independently to increase reliability. Coding disagreements were adjudicated through discussion between LJ and MY. If disagreements could not be resolved through discussion, then TJ would make a decision. However, this was not needed as all discussions led to resolutions for the coding and TJ agreed with the coding decisions.

The codebook was developed iteratively in each round. We developed the codebook on three interview transcripts and then added three more transcripts to test the codebook, through which we identified new codes. We then returned to the original three transcripts and recoded with the revised codebook. This process ensured that codes were saturated. From the coding, we developed a grounded theory based on patterns in the coding and the associations between these codes. Construct validity was achieved through constant comparison and member checking. Internal validity was achieved through diversity within the research team. Data analysis was supported by NVIVO 12.0 software.

### Positionality and reflexivity statement

Our positions have informed the way we have interpreted the data underpinning the proposed grounded theory.

Linya Jin is a native Chinese speaker and is completing her doctoral research in China concerning prosocial behavior and professionalism education for medical students. She has no prior relationship with any of the research participants.

Tanisha Jowsey is a New Zealand pakeha with 25 years of qualitative research experience, including experience of conducting grounded theory research. She is currently an associate professor of medical education and professionalism in Australia and has no prior relationship with any of the research participants. Tanisha is a native English speaker. She has research experience in these relevant fields: medical education, empathy, altruism, and Confucianism.

Mei Yin, a native of China, is now a professor of medical humanities education and has 34 years of teaching and research experience in medical education. She has no prior relationship with any of the research participants.

### Ethical approval

As we recruited participants from all over the country, the interviews were conducted online rather than face-to-face. Due to the format of the interviews, verbal informed consent was used in this study. Prior to the interview, we read the full informed consent form and the interview outline. All participants gave verbal consent to the above before the start of the formal interview. The Institutional Research Board of Harbin Medical University approved the verbal informed consent and the research proposal. (IRB reference number: HMUIRB2023022.)

## Results

Twenty-eight medical students participated in this study. Twenty-three participants identified as female (82.1%) and five as male (17.8%). Twenty-one participants were undergraduates, and seven were postgraduate medical students. Participants were aged 20–25 years, with an average age of 22.1 years. Participants came from one of 21 universities across 15 states in China. Table 1 shows the demographic characteristics of all participants.

**Table 1:** Demographic characteristics

Demographic information (numbers expressed as percentages and (n))	
Mean age (years)	22.1
Male	17.8 (5)
Female	82.1 (23)
Specialty	
Clinical Medicine	46.4 (13)
Pediatrics	10.7 (3)
Anesthesiology	7.1 (2)
Nursing	7.1 (2)
Medical Laboratory	7.1 (2)
Preventive Medicine	3.5 (1)
Biomedical	3.5 (1)
Dentistry	7.1 (2)
Traditional Chinese Medicine	7.1 (2)
In China, undergraduates start choosing a major and working towards it from their first year of study	
Region	
North China	17.8 (5)
East China	14.2 (4)
Northeast China	28.5 (8)
Central China	3.5 (1)
South China	17.8 (5)
Southwest China	3.5 (1)
Northwest China	14.2 (4)

After analyzing the interview data of factors influencing the prosocial behavior of medical students, the open codes were divided into 14 subcategories through the axial coding process, and these subcategories were divided into six overarching categories: personal values, personality traits, perceived self-competence, career motivation, environmental factors, and family influences. Table 2 describes the thematic elements of the major categories and subcategories. From these major categories and subcategories, we arrived at a grounded theory of medical student views of prosocial behavior.

**Table 2:** Overview of all identified categories and subcategories from participant interviews

Categories	Subcategories	Themes
Personal values	Self-Transcendence value	• Benevolence: sense of meaning, sense of value, helpfulness, responsibility • Universal values: fairness, equality
	Conservation value	• Self-protection • Risk perception
	Socialist value	• Chinese core socialist values
Narrow personality traits	Empathy	• sympathy • put oneself in someone else's shoes
	Resilience	• Self-regulation • Positive mindset
	Moral sensitivity	• Awareness • Knowing a better way to do things
	Mutual help and reciprocity	• Positive motivation • Goodwill transfer, positive circulation
Perceived self-competence	Perceived self-competence	• Experience • Knowledge required for behavior
Career motivation	Social and altruistic motivation	• To save lives and help the sick and injured • Career pursuit • Passion for medicine • Helping others
	Money and prestige motivation	• To provide for the family • Economic motivation • performance appraisal • Work within the division • Development and promotion
Environmental factors	School environment	• Prosocial activities • Humanistic education • Role model education
	Organizational environment	• Policy development • Occupational stress
Family influences	Family education	• Teach by word • Teach by example
	Family atmosphere	• Family support • Caring for each other

### Medical student prosocial behavior grounded theory summary

The theory we propose, based on student voices, is that prosocial behavior among medical students is influenced by a complex interplay of personal values, narrow personality traits, perceived self-competence, career motivation, environmental factors, and family influences. Personal values, including self-transcendence values such as benevolence and universal values like fairness, drive intrinsic motivation for prosocial behavior. Socialist values, influenced by external factors, also play a role. Narrow personality traits such as empathy, resilience, and moral sensitivity contribute to individuals' propensity for prosocial behavior. Perceived self-competence reflects individuals' judgment of their ability to engage in prosocial acts. Career motivation, driven by social and altruistic motives or monetary and prestige motives, shapes individuals' orientation towards prosocial behavior. Environmental factors, such as school and organizational environments, and family influences further shape individuals' perceptions and behaviors towards prosocial acts. In the following section, describe the theory with reference to our study participants and what they said, as well as other relevant social theories.

### Medical students' perceptions of prosocial behavior

Participants demonstrated they had a clear concept of prosocial behaviors. In Smith’s Theory of Moral Sentiments, he distinguished between "ordinary" prosocial behavior and " extraordinary" prosocial behavior [29]. Ordinary prosocial behaviors involve relatively high situational and sociocultural demands that are explicit in their needs. In contrast, extraordinary prosocial behaviors involve relatively low contextual and sociocultural demands, and the need for these behaviors is sometimes ambiguous [30]. As with Smith’s Theory of Moral Sentiments, participants in this study focused on "ordinary" prosocial behaviors. That is, they discussed the prosocial behaviors of medical students that arise from specific roles, concepts of professionalism, and scopes of practice associated with medical practice. Participants described diverse prosocial behaviors that they had undertaken (listed in Table 3).

**Table 3:** Self-reported behaviors that medical student participants identified as prosocial

• Blood donation
• Protecting the welfare of laboratory animals
• Volunteering for medical care
• Collecting donations for patients in financial need
• Performing resuscitation in emergency situations
• Online and offline health education
• Caring for patients
• Improving doctor-patient communication
• Non-profit medical aid

Participants also mentioned altruistic behavior. Altruistic behavior is often mentioned listed as a professional attribute of doctors. Editors of the New England Journal of Medicine (Editorial 2000) describe medical practice as ''unambiguously'' altruistic [16]. However, participants in this study did not see themselves as necessarily altruistic. Instead, they saw themselves benefiting from engaging in medical practice, conjuring up concerns of moral abduction and role pressure.

*I think “altruism” and “egoism” are opposites, and all the actions I do are done because I feel I get something out of them, like learning, getting psychological satisfaction, solving my own problems, etc. I always thought I was helping others and then getting something from them, and that would not be purely altruistic behavior. So, I feel that there is a part that is good for me, then my behavior is not altruistic. And altruism contains an implication of self-sacrifice, which I most likely won’t do. (Yan, Participant 23)*.

Medical students were also interviewed about their perceptions of current medical humanities education and its role in prosocial behavior. Participants believed that prosocial behavior is necessary for practitioners in the medical profession, but there needed to be more certainty about how prosocial behavior is motivated and the role of current medical education in promoting prosocial behavior among medical students. This is partly because prosocial behavior depends on the values, personality traits, and life experiences that individuals possess before they decide to study medicine. However, although basic prosocial competencies vary, there are specific ways in which medical students can trigger and motivate the emergence of prosocial behaviors during medical school studies and hospital internships. Medical students suggest that,

*Going into each specific patient’s story and sharing the patient’s treatment and post-illness journey allows me to better understand the illness from the patient’s perspective, understand and experience the impact of the illness on the patient and family, and improve my ability to think differently than learning medical humanities content in the theoretical classroom. (Jie, Participant 3).*

*Exposure to concrete cases during clinical practice is more likely to trigger prosocial behavior than learning in the theoretical phase. (Lin, Participant 4)*

### Factors influencing prosocial behavior

#### Personal values

Participants identified self-transcendent values and socialist values as the driving forces behind their prosocial behavior. Participants described self-transcendent values such as benevolence, a sense of meaning or purpose, value, responsibility, and fulfillment. These are intrinsic motivating factors.

*I think the realization of my self-worth is that I can help others, which I think is a very sacred thing. I help people and I’m happy to see them happy, even if the thing doesn’t do me any good. I hope that everything I do is done with a clear conscience and is beneficial to society or others. (Qi, Participant 19)*.

Participants also described universal values driving their prosocial behavior, such as fairness and equality.

*Especially during times of high epidemic, when hospitals do not have enough beds and medical staff, many patients will go to doctors and nurses for help, and when faced with this dilemma, an inequity is triggered. Medical staff themselves may also have difficulty choosing when making choices, not knowing exactly who to help. I hope I can do something modest to alleviate this situation. (Bo, Participant 15).*

Chinese core socialist values (extrinsic motivating factors) were also mentioned by several participants, who identified socialist values as potential facilitators of prosocial behavior.

*I’m a party member, so I just think it’s very meaningful to do these things, and this could be a kind of dedication. (Quan, Participant 7)*

Participants described complexity in their perceptions of prosocial behavior as entailing potential risks to their sense of security. When participants perceived a potential threat to their security associated with prosocial behavior, they said this would deter them from engaging in prosocial behavior (focusing on their own rather than others’). For example, participants perceived tensions in doctor-patient relationships and medical risks associated with care as potential spaces in which they needed to tread carefully, and this was considered within the broader context of their internship. They described needing to manage risk to complete their internship successfully.

*Some teachers also told me that you should not be too nice to the patient, you should not empathize with the patient, you should keep a certain distance from the patient, your kindness can sometimes harm you, you have to learn to protect yourself. (Wen, Participant 5).*

*Doctors are indeed a very difficult profession, always in the midst of a high-risk environment. It is possible that the next patient will have some overreaction, so you need to always ask yourself to be on edge. (Chen, Participant 14).*

### Narrow personality traits

This category refers to individuals’ internal features and can be defined as relatively stable patterns of emotion, motivation, cognition, and behavior.

#### Empathy

Empathy refers to one individual’s reactions to the observed experiences of another [31]. Study participants mentioned ‘clinical empathy’, which is an affective and cognitive understanding of the patient’s reactions, thoughts, or feelings, such as sympathizing with others, empathizing with others, and putting oneself in someone else's shoes and then demonstrating understanding to the patient in a behavioral manner.

*When I see life and death in the hospital, when I see the patients and their families in a very sad, worried and uncomfortable state, I feel that my heart also becomes distraught with them. (Wei, Participant 1).*

#### Resilience

Resilience is the successful adaptation of adversity and environmental stressors [32]. People with high resilience show prosocial tendencies.

*I think I am still more optimistic, even if I have encountered some unfriendly situations during my internship, most of the people are definitely still good, and I can still go to keep an optimistic mind to face my study and life. Once during my internship, I saw a rather sad patient and I cried at night thinking about it. But my friend said to me, "You can be sad, but you can't stay sad." I kept using that quote to help me come out of it quickly and think about what I could do to help in a substantial way. (Chao, Participant 18)*.

#### Moral sensitivity

Medical students reported that they recognized situations during their clinical clerkships that had moral problems or moral dilemmas and that they knew some better ways to respond to these problems. This formulation fits with the concept of moral sensitivity. Moral sensitivity is the ability to identify the existing moral issue. It represents the process by which individuals perceive the presence of an ethical dilemma, understand moral situations, and make the right ethical decision on taking the appropriate measures [33].

*I saw my lead instructor, and he was very indifferent to a patient, and I realized at the time that that was not good. Well, it's just that in many cases, it's actually hard for you to say. (Cheng, Participant 16).*

*In the dermatology internship, the lead teacher was just very mechanical, his focus was on the students and not on the patients. I watched him on his rounds, just being very cold and icy, like a piece of wood, like a piece of ice. I don't think it should be that way, how busy is it? Maybe he has a lot of patients in his hands, but the patient has only one doctor, so I think it is better to pay more attention to the patient. (Ge, Participant 25)*

### Mutual help and reciprocity

Reciprocity states that people should help those who have helped them and avoid injuring those who have helped them [34]. Medical students expressed two types of reciprocity situations, direct and indirect reciprocity, when sharing motivations for prosocial behaviors that they had done in the past. Direct Reciprocity (DR) means that the individuals reciprocate acts to the giver [35].

*The patient was anxious himself because he needed to change his medicine for a fracture. He saw my badge and knew I was a student, but he was cooperative with each change and gave me encouragement to practice more and study better. I was touched by his willingness to let me grow and became more concerned about his situation. (Xuan, Participant 8)*.

Indirect reciprocity examines individual decisions to reciprocate acts of kindness to strangers when individuals are the beneficiary of someone else's acts of kindness [35]. Medical students are willing to help others but expect others (not necessarily the same ones) to help them if needed.

*I think prosocial behavior will definitely bring positive feedback. Maybe if I help others today, others will help me one day. (Quan, Participant 7)*

*I am a little superstitious, I believe that doing good deeds can accumulate some luck. It's like I give to others, then others may not feedback to me, but he feedback to others, and then this kindness will eventually turn back to me. (Qi, Participant 19)*

### Perceived self-competence

The self-perception of competence reflects people's judgment of their own abilities to mobilize resources to achieve a particular goal [36]. During the interviews, multiple participants mentioned that they may not engage in prosocial behavior because they do not think they have the knowledge or skills needed.

*Some older professors are more experienced, understand more, and are more likely to care for and help others. He has a better way of communicating with students and patients, and he is able to grasp the psychology of the people he is communicating with.* *(Wei, Participant 1)*

*I feel like my senior year, with more knowledge, I'll be more empowered to take action. I am also more capable of giving people health science and helping them improve their health. Freshman and sophomore year I felt like I wasn't capable of doing that. (Sheng, Participant 21).*

### Career motivation

Career motivation is a multidimensional concept organized into three prior domains: career resilience, insight, and identity [37]. The career motivation theory is used to understand career-related attitudes and behaviors, including patient-related prosocial behaviors. Participants mentioned two career motivations for choosing to pursue a career in health care: social and altruistic motivations、money and prestige motivations.

### Social and altruistic motivation

Participants described social and altruistic intrinsic motives as conducive to prosocial behavior. Such motivations include saving lives and helping sick and injured, career pursuit, passion for medicine, and helping others.

*A doctor, if he is out of the heart to choose to study medicine, he must have a real desire to cure people's mindset, really want to save lives and help the injured. In this case, he will certainly be enthusiastic, with goodwill, do his best to help the patients. (Cheng, Participant 16)*.

*Studying medicine is a sentiment, an ideal to make this a lifelong career. I treat it as a lifelong career or a passion in doing it. (Rui, Participant 24)*

*Because I have wanted to study medicine since I was a child, I have a great passion for it. I would like to put more energy into it and study more things, including paying more attention to the details of communication and patient care, and helping as many people as possible. (Ting, Participant 22)*.

### Money and prestige

Participants described other students as driven by money and/or prestige to enter the medical profession. Few participants described holding this motivation themselves. When they did, some participants described the income associated with medical practice as ensuring they could continuously provide for their family’s needs. There is an altruistic element in this perspective.

*Some people study medicine to make money, to put it bluntly, to support the family. Medicine and health industry salary level is okay, more stable, do not make any big mistakes, it is also considered an ‘iron rice bowl’ job. (Yu, Participant 6)*.

Participants described other students/doctors as simply a means to an end.

*Some people do not like the profession of medicine, he will think that as long as the job can be done, there is no higher level of spiritual pursuit, to be a doctor may be the same as to be a restaurant waiter. (Jie, Participant 3)*.

Participants noticed that their accrediting bodies and workplace employers do not reward prosocial behavior. That is, workers will be paid the same regardless of whether they engage in prosocial behavior.

*Before becoming a doctor, part of the students are profit-oriented, profit maximization, they believe that what is good for me, I just go to do, and do not care about the feelings of others. (Rui, Participant 24)*.

*These prosocial behaviors will also not be included in the performance review. My caring for patients or not caring will not affect my annual review, then I don't need to do it. (Xin, Participant 10)*.

### Environmental factors

#### School environment

Participants observed that medical curricula included prosocial activities and humanistic education. They also noticed the prosocial behavior of their teachers and clinical supervisors. Participants valued formal and informal learning opportunities concerning prosocial behavior in clinical practice.

*From the time we entered medical school, we were taught the Hippocratic Oath, and we were told in class that in addition to treating patients, doctors must also provide humanistic care to patients and take into account their family situation and their financial problems. (Sheng, Participant 21)*.

*When we were learning first aid, our teachers were very firm in telling us that you, as a medical student, you cannot turn a blind eye or just make an emergency call when you encounter an emergency situation, you must do what you can. (Ting, Participant 22)*.

Role modeling is a core element in student learning during the clinical internship stage of medical education. Participants described this largely hidden curriculum as pivotal to shaping student ideas about prosocial behavior.

*The education we receive in the hospital can be positive or negative, depending on what teachers we encounter. If I enter a very fast-paced department, or if I come into contact with teachers who are indifferent or numb, then I may also become indifferent. I have met teachers in the internship who are particularly warm to patients, and my behavior is subconsciously influenced. (Ting, Participant 22)*.

#### Organizational environment

The policies of healthcare organizations also have a significant effect on guiding the behavior of individuals.

*Because our hospital has relatively strict rules on the issue of doctor-patient relations, patients who go to complain about you because of your bad attitude are subject to deductions, and I think a lot of prosocial behavior in the hospital setting is based on this policy, but it may have the opposite effect. (Chao, Participant 18)*.

And some inappropriate policies can inhibit individuals' prosocial behavior.

*During the epidemic, those nurses went to support all over the motherland and had to be docked money after they turned out to be positive and had to insist on working with illness, which I thought was all impossible to happen, but it just happened and it made me feel frustrated. (Lian, Participant 12)*.

Another factor that has a significant impact on prosocial behavior in healthcare organizations is occupational stress; participants mentioned the quest for efficiency, fatigue, research pressure, and competitive pressure. Participants observed that,

*Some doctors don't explain too much to patients because medicine is too complicated and doctors are in a hurry to achieve their goals in the pursuit of efficiency. They want the patient to sign in a hurry and then rush the surgery. (Long, Participant 13*).

*Doctors are probably very tired and no longer have the energy to care compassionately for others. (Xin, Participant 10)*

### Family influences

The final theme that we identified concerns the role of family on participants’ views concerning prosocial behavior. Participants described family education and support as significantly associated with prosocial behavior in that family context shaped participants’ formative thinking and experiences concerning prosocial behavior. Participants interpreted their family context as shaping their personality and values before entering medical school. This influence cannot be understated.

*My grandmother was an old Chinese medicine doctor, and I could see how she lived her life, how she combined her knowledge of Chinese medicine in her life, how she dealt with people and how she treated her patients. I was influenced by her subconsciously. (Chen, Participant 14)*.

*My family cared for each other and took care of each other when I was growing up, so naturally I would care for others. But some people have not experienced a lot of care and no one to take care of him, he may be less able to care and help others, and he may not know how to care for others at all. (Xin, Participant 10)*.

## Discussion

This study examined medical students' perceptions of prosocial behavior and its related concepts and supported the use of prosocial behavior rather than altruism as a professional value in medical practice. One element within the Theory of Moral Sentiments is relevant to our study: *Section II of Justice and Beneficence.* Smith differentiated between the virtues of justice and beneficence, the former meaning fulfilling an obligation properly and the latter indicating benevolent behaviors such as generosity, charity, kindness, and friendship [29]. Medical students in our study had a relatively straightforward perception of prosocial behaviors. They viewed prosocial behaviors as critical elements of professionalism and supportive of a stable/harmonious healthcare industry. Smith identified prosocial behavior as benevolent. Whereas our participants conceptualized prosocial behavior as obligatory. This fits with the theory of planned behavior. The theory of Planned Behavior states that attitudes are a prerequisite for motivation, which is related to certain behaviors. According to the Theory of Planned Behavior, attitudes are perceptions of a certain behavior, including evaluating the behavior [38]. Medical students have a more open attitude towards prosocial behaviors than altruistic ones. Previous research has also shown that altruism in medicine is at least declining, if not dying out [39]. This may be related to the general uncertainty of altruism and the risk of burnout due to its association with self-sacrifice [40]. This instability supports our proposal to shift the focus from "altruism" to "prosocial behavior" in medical professionalism education, which is consistent with the views of some scholars [16, 17].

**Fig. 1:**
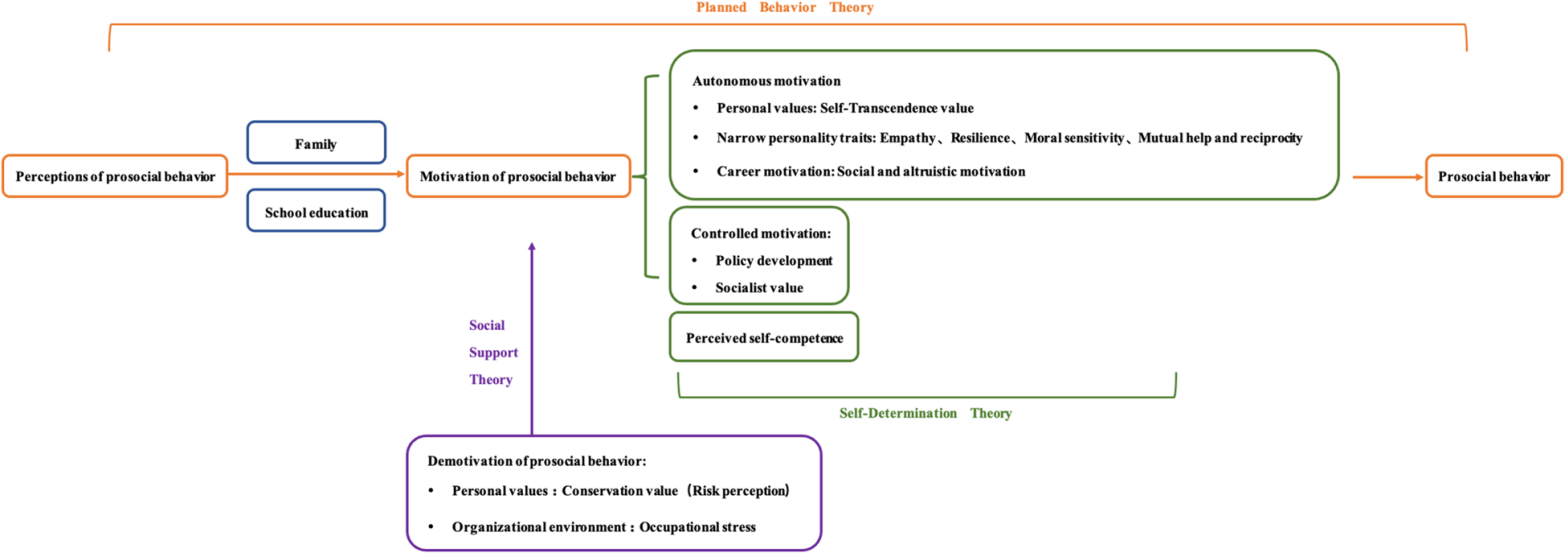
Grounded theory of what governs medical student prosocial behavior

Based on the analysis of factors influencing the prosocial behavior of medical students, this study identifies which factors contribute to the motivation or demotivation of prosocial behavior (see Fig. 1). Our findings are by and substantiated by the Self-Determination Theory. Self-determination theory proposes the fulfillment of the three basic psychological needs of autonomy, competence, and relatedness as a central concept. Autonomy, in turn, divides behavioral motivation into intrinsic and extrinsic motivation [41]. Prosocial behavior can be driven by personal values and initiatives (intrinsic motivation) and by external pressures or rewards (extrinsic motivation) [42]. In this study, the intrinsic motivations of medical students included self-transcendence values, personal traits, and professional motives. These motivations can be fostered through family contexts and in medical school curricula. External motivations such as money and prestige should be considered carefully by policymakers and healthcare institutions. An initial question is: given that student doctors readily identify behavior drivers in policy and practice, should healthcare institutions address prosocial behavior specifically in policies and remediation drivers? At the same time, medical school education and training also affect medical students' perception of self-competence, and there is a need to increase the teaching of specific knowledge and skills related to prosocial behaviors and to enhance relevant practical training to improve the perception of self-competence of prosocial behaviors and the perception of relatedness of medical students.

Demotivation of prosocial behavior is closely related to the risk perception in conservation value and occupational stress in the organizational environment. This study suggests that social support theory may ameliorate demotivation. Social support refers to psychological help or material support such as care, respect, and meeting needs from family members, friends, organizations, and other members of society [43]. Social support is an important personal resource closely related to prosocial behavior [44]. Medical students' perceived occupational risk and stress can be alleviated through organizational management and social support. This study suggests that providing prosocial literacy training for medical students in the "hidden curriculum" or "clinical practice" may be more effective. Previous studies have also made relevant findings that medical students want to be taught ethics in practice and to develop their ethical practices, rather than being taught ethics [45].Developers of educational programs and administrators in the healthcare industry can use and validate the model developed in this study in their future endeavors, as this study provides preliminary information for promoting prosocial behaviors among medical students.

Previous studies have shown that the prosocial behavior of college students is related to personal traits, family factors [46], environmental factors [47], and values [48]. This study shows that medical students also have a significant effect on career motivation and prosocial behaviors compared to college students in other majors. In addition, we analyzed the motivation or demotivation of prosocial behavior of medical students based on the factors influencing prosocial behavior and established a model of prosocial behaviors of medical students based on the Theory of Planned Behavior, Self-Determination Theory, and Social Support Theory, which was recommended to be promoted through family education, school education. This helps to identify possible interventions for the prosocial behavior of medical students in medical training (Fig. 1).

The current study has some limitations. We used a convenience sampling method, which may have resulted in a biased sample that does not represent the broader population. As a result, the findings of this study may not be generalizable across all regions of China or abroad.

## Conclusions

We explore medical students’ understanding of prosocial behavior and the factors that influence their engagement in prosocial behavior in China. From the participants’ voices, we developed a grounded theory. We posit that medical students hold especial views on the roles of medical doctors, and the majority of these views align with student core values, including the value of prosocial behavior. Students are intrinsically motivated to engage in prosocial behaviors that align with their core values. This study supports a focus on prosocial behavior instead of altruistic behavior in medical education.

### Supplementary Information

**Supplementary Material 1.**

## Data Availability

The data used and/or analyzed during the current study are available from the first author on reasonable request.
