# Correction: Medical students’ perceptions of prosocial behaviors: a grounded theory study in China

**DOI:** 10.1186/s12909-024-05418-x

**Published:** 2024-04-22

**Authors:** Linya Jin, Tanisha Jowsey, Mei Yin

**Affiliations:** 1 School of Medical Humanity, Harbin Medical University, Nangang District, Harbin, Heilongjiang Province, China; 2 School of Medicine, Bond University, 14 University Drive, Robina, Gold Coast, QLD, 4226 Australia

**Correction: BMC Medical Education (2024) 24:353**

10.1186/s12909-024-05335-z

Following publication of the original article [1], the authors informed us that accidentally deleted a paragraph which describes the grounded theory.

The original article has been corrected.
