# Supplementary material for: Medical students’ perceptions of prosocial behaviors: a grounded theory study in China
Source: BMC Med Educ. 2024 Mar 29;24:353. doi: 10.1186/s12909-024-05335-z (PMC10981338; doi:10.1186/s12909-024-05335-z)
Supplement: Supplementary file 1 — Supplementary Material 1. [file 12909_2024_5335_MOESM1_ESM.docx]

| **Appendix 1 Interview guiding questions** |
| --- |
| 1. Talk about your understanding of pro-social behavior and altruistic behavior 2. As a medical student, what are the pro-social behaviors that you have observed in your life and study process? 3. As a medical student, have you ever engaged in pro-social behavior in your life? 4. What helped you to behave in this way? 5. Have you ever engaged in pro-social behavior with patients during your clinical practice? 6. Under what circumstances were you more likely to engage in behavior that was more beneficial to the patient? 7. Based on your observations or personal experience, what could be a barrier to engaging in pro-social behavior? 8. Do you think that current humanistic education helps to develop pro-social behavior in medical students? 9. What lessons or moments in medical school have motivated you to engage in pro-social behavior? |
